# Bioinspired ultra-stretchable and anti-freezing conductive hydrogel fibers with ordered and reversible polymer chain alignment

**DOI:** 10.1038/s41467-018-05904-z

**Published:** 2018-09-04

**Authors:** Xue Zhao, Fang Chen, Yuanheng Li, Han Lu, Ning Zhang, Mingming Ma

**Affiliations:** CAS Key Laboratory of Soft Matter Chemistry, iChEM (Innovation Center of Chemistry for Energy Materials), Department of Chemistry, University of Science and Technology of China, Hefei, Anhui 230026 China

## Abstract

High-performance stretchable conductive fibers are desired for the development of stretchable electronic devices. Here we show a simple spinning method to prepare conductive hydrogel fibers with ordered polymer chain alignment that mimics the hierarchically organized structure of spider silk. The as-prepared sodium polyacrylate hydrogel fiber is further coated with a thin layer of polymethyl acrylate to form a core–shell water-resistant MAPAH fiber. Owing to the coexistence and reversible transformation of crystalline and amorphous domains in the fibers, MAPAH fibers exhibit high tensile strength, large stretchability and fast resilience from large strain. MAPAH fiber can serve as a highly stretchable wire with a conductive hydrogel core and an insulating cover. The stretchability and conductivity of the MAPAH fiber are retained at −35 °C, indicating its anti-freezing property. As a prime example of stretchable conductive fibers, MAPAH fibers will shed light on the design of next generation textile-based stretchable electronic devices.

## Abstract (web summary)

High-performance stretchable conductive fibers are desired for the development of stretchable electronic devices but preparation of conductive hydrogel fibers is challenging. Inspired by spider silk the authors demonstrate here a spinning method to prepare stretchable conductive hydrogel fibers with ordered polymer chain alignment.

## Introduction

Stretchable conductive materials are essential for the emerging stretchable electronic devices^1,2^, such as stretchable panels^3^, energy-storage devices^4–6^ and sensors^7,8^. Among various functional hydrogels^9–11^, conductive hydrogels are very promising as stretchable conductive materials, which have been extensively studied for applications such as stretchable sensors^7,8,12^ and supercapacitors^13,14^. Most of the conductive hydrogels are made into two-dimensional films or three-dimensional monoliths by molding methods^15,16^, which result in an amorphous material with polymer chain’s random orientation and disordered alignment. If conductive hydrogels can be made into one-dimensional fibers with ordered alignment of polymer chains, some properties of conductive hydrogel fibers (e.g., mechanical properties and conductivity) would be greatly enhanced over conventional conductive hydrogel films and monoliths^17^. However, the ordered chain alignment of conductive polymers has been achieved in solid-state microfibers prepared by template directed polymerization^17–19^ or alignment^20^, which are not suitable for the preparation of conductive hydrogel fibers at macroscopic scale. On the other hand, spinning methods are widely used to prepare polymer fibers with ordered chain alignment^21,22^. But conductive hydrogels are rarely made into long fibers by spinning, due to the poor spinnability of current conductive hydrogels or their precursor solutions. In addition, conductive hydrogel films and monoliths are also limited by their slow resilience from large deformation due to the moderate reversibility of polymer chain alignment^11^, and the loss of functions at subzero temperature^23^. Previously reported polyelectrolyte fibers based on polycation–polyanion interfacial complexation are rigid and insulating materials, which are mainly used for biomedical applications, such as tissue engineering and drug delivery^24,25^. Previously reported stretchable conductive fibers based on carbon materials^6,26^ or metal nanomaterials^27,28^ coating on elastomeric fibers are also limited by their sophisticated fabrication process and moderate stretchability^29^. Therefore, high-performance conductive hydrogel fibers with ordered and reversible chain alignment are highly desired for the development of stretchable electronics, especially for the textile-based stretchable electronic devices^14,30^, but remain as a challenge.

In nature, spiders spin silk fibers from aqueous protein solutions at ambient conditions^31^. The hierarchically organized structure of spider silk^32^ and its unique spinning process^33^ are the key factors to achieve its superb properties^34^. For example, spider dragline silk is a semi-crystalline protein polymer, where alanine-rich crystalline regions are connected by soft glycine-rich amorphous regions as linkers^32^. Inspired by the organized structure and the unique spinning process of spider silk, we propose to develop a simple spinning method to prepare conductive hydrogel fibers with ordered and reversible chain alignment from aqueous solution of polyelectrolytes at ambient conditions. Aiming at the production at low cost, a mass-produced synthetic polyelectrolyte: sodium polyacrylate (PAAS), was chosen as the starting material, which has been widely utilized in daily supplies and in industry. In water solutions, the anionic carboxylate groups on a PAAS chain repel each other via double layer forces, which causes the polymer chain to adopt an expanded, rigid-rod-like conformation. Adjusting pH, addition of salts or poor solvents tune the electrostatic interactions and alter the conformation of PAAS chains in solution, leading to the change of bulk properties (such as viscosity and sol–gel transition)^35^. Based on the knowledge learned from spider silk^31–33^, we expect that the stimuli-responsive properties of PAAS in solution would enable PAAS solution a good spinnability, where the spinning and drawing process would enable an ordered alignment of PAAS chains in the as-spun filaments.

After a systematic exploration, we have found that long and uniform filaments can be readily drawn from a gel-like PAAS solution in a mixture of water (as a good solvent) and dimethyl sulfoxide (DMSO, as a poor solvent). Water evaporation from the PAAS filaments in air enriches the poor solvent DMSO in the filaments, which triggers a quick phase transition to form PAAS hydrogel (PAH) fibers with adjustable diameters and arbitrary length as wish. Just like spider silk, PAH fiber shows a unique beads-on-a-string structure^36^ and good mechanical properties. To achieve a good water resistance property, PAH fibers are coated with a thin layer of polymethyl acrylate (PMA) to form core–shell PMA–PAAS hydrogel (MAPAH) fibers. Remarkably, the water-resistant MAPAH fibers exhibit a unique combination of high tensile strength (5.6 MPa) and stretchability (elongation at a break of 1200%), fast resilience (<30 s) from large stretching strain, high electrical conductivity (2 S m^−1^), and great anti-freezing property. The simultaneous achievements of these prime properties by MAPAH fibers are attributed to the coexistence and reversible transformation of crystalline and amorphous domains in hydrogel fibers, which are enabled by the spinning and gelation process. The multifunctional MAPAH fiber as a high-performance and low-cost stretchable conductive fiber will shed light on the design of next generation textile-based stretchable electronic devices.

## Results

### Fabrication of PAH and MAPAH fibers

**Fig. 1:**
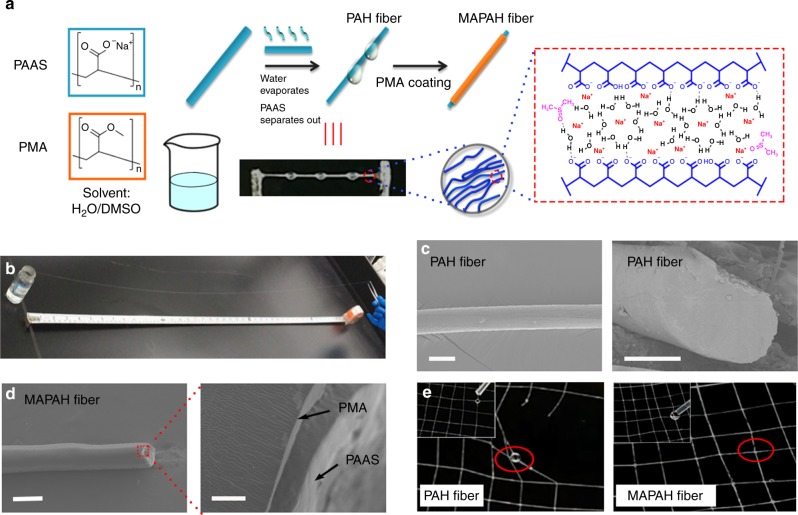
Preparation of PAH and MAPAH fibers. **a** Schematic illustration of the preparation of PAH and MAPAH fibers. A photograph of as-prepared PAH fiber shows the beads-on-a-string structure. **b** Photograph of a 1.1 m-long as-prepared PAH fiber. **c** SEM images of a PAH fiber. The scale bar is 100 μm. **d** SEM images of a MAPAH fiber. The scale bar in the left image is 200 μm, and the scale bar in the right image is 5 μm. A thin PMA layer on the PAAS core is clearly observed in the right image. **e** Photograph of a PAH web damaged by liquid water and a MAPAH web resistant to liquid water. Red circles indicate water droplets remaining on the hydrophilic PAH fiber, while no water droplet stays on the hydrophobic MAPAH fiber

Among many different types of commercial available PAAS, the non-crosslinked PAAS with an ultra-high molecular weight (*M*_w_ ~ 3 × 10^7^ Da) and low cost (~$20 per kilogram) was chosen as the starting material. The preparation procedure of PAH and MAPAH fibers is summarized in Fig. 1a. PAAS powder was dissolved in a mixture of water and DMSO at 80 °C to form a transparent solution (Supplementary Fig. 1). The composition of the water/DMSO mixture and the concentration of PAAS in the solution are the two key factors for the successful preparation of PAH fibers. The optimal solution was found as 4 wt% PAAS dissolved in the water/DMSO mixture solvent with DMSO% = 20 wt%. As shown in Supplementary Fig. 2, 4 wt% PAAS solutions in the water/DMSO mixture with different water: DMSO ratios were prepared at 80 °C. Upon cooling to room temperature, a clear phase transition process was observed, indicating a critical concentration of DMSO in the range of 20–22 wt%. With DMSO% ≤20 wt%, the PAAS solution remained as a homogeneous solution at room temperature. With DMSO% ≥22 wt%, a white precipitate gradually formed during the cooling process. A similar phase transition was observed for covalently crosslinked PAAS hydrogels swelled by water/DMSO mixture^37^. The addition of DMSO into water causes significant desolvation of PAAS chains upon the counterion Na^+^ binding carboxylate groups, increasing the polymer–polymer interactions and triggering the phase transition^37^. The optimal solution for PAH fiber preparation has DMSO% = 20 wt%, which is very close to the critical DMSO concentration for the phase transition. Therefore, when a PAAS filament was drawn out from the optimal PAAS solution at room temperature, the DMSO% in the filament increased due to water evaporation and quickly exceeded the critical concentration. Therefore, PAAS in the filament rapidly aggregated to form a fine PAH fiber, while the excess aqueous solution can automatically form liquid droplets due to surface tension (Fig. 1a). These liquid droplets either flowed away along a tilted PAH fiber (Supplementary Movie 1) or gradually solidified on a horizontal PAH fiber to form the beads-on-a-string structure that mimics spider silk^30^ (photograph in Fig. 1a). Similar to spider silk, PAH fibers are elastic and sticky, which can be used to weave a web that mimics a spider web (Supplementary Fig. 3). PAH fibers with arbitrary length can be easily made through continuous drawing from the optimal PAAS solution. Two examples of as-prepared PAH fibers are shown in Fig. 1b (~1.1 m) and in Supplementary Movie 1 (~6.2 m). Since the preparation of PAH fibers is similar to the process of gel spinning^21^, we envisage that the preparation could be facilely adapted onto a gel spinning equipment for the mass-production of PAH fibers.

Due to the hydrophilic nature of PAAS, PAH fibers can be quickly swelled and weaken in liquid water. To achieve a good water resistance, we coated the PAH fibers with a thin hydrophobic layer of polymethyl acrylate (PMA). PMA was synthesized through conventional radical polymerization (*M*_*n*_ = 3.8 × 10^5^ Da, PDI = 1.93, see SI for details). As-prepared PAH fibers were immersed in a 5 wt% PMA solution in ethyl acetate for several seconds and then taken out. After the quick evaporation of ethyl acetate, a thin and uniform layer of PMA was firmly coated on the PAH fiber to form the core–shell MAPAH fiber. As judged by scanning electron microscopy (SEM, Fig. 1c, d), both PAH and MAPAH fibers show a cylindrical shape with consistent diameter. Observed under an optical microscope, both fibers show a good uniformity of their diameter along the fiber length (Supplementary Fig. 4). Fracture surfaces from both fibers show a compact and homogenous interior core. As shown in Fig. 1d, a thin and compact PMA layer can be clearly observed on the PAH fiber surface that firmly wraps the PAH core. PAH fibers with different diameters in the range of 20–300 μm can also be prepared by varying the preparation conditions (Supplementary Fig. 5), which can be further converted to MAPAH fibers. Although the thickness of PMA layer was only 1–2 μm, this compact PMA layer enables MAPAH fibers a great water resistance property. Observed under an optical microscope, a PAH fiber was swelled and gradually dissolved by a water droplet, while the MAPAH fiber remained unchanged with the water droplet (Supplementary Fig. 6). The contact angle of water droplet on MAPAH fiber was measured as ~92° (Supplementary Fig. 7), indicating a good hydrophobicity of the PMA coating layer. As shown in Supplementary Movie 2, a MAPAH fiber retained its great water resistance property even in a stretched condition (~200% elongation), while a PAH fiber was quickly broken by liquid water at the same condition. As expected, the web made from PAH fibers was not resistant to liquid water, while a water-proof web can be made from MAPAH fibers, which remained stable and elastic after being treated by liquid water (Fig. 1e).

### Characterization and optimization of the PAAS solutions

**Fig. 2:**
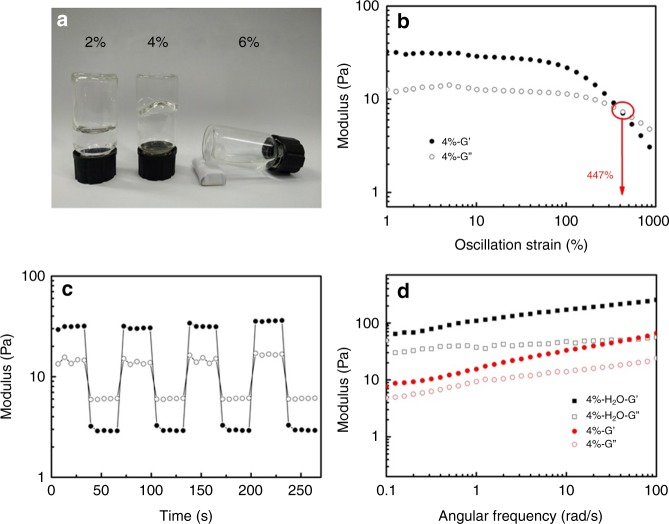
Preparation and rheological characterization of PAAS solutions. **a** Photograph of PAAS solutions with different concentrations (2%: viscous liquid; 4%: physical gel; 6%: precipitate and liquid) in H_2_O:DMSO = 4:1 mixture at room temperature. **b** Strain-dependent and **c** step-strain oscillatory rheology of the optimal PAAS solution. The applied oscillatory strain in **c** alternated between 1 and 1000% for 30 s periods (*ω* = 10 rads^−1^, 25 °C). **d** Frequency-dependent oscillatory rheology of 4% PAAS solution with pure water (black) or H_2_O: DMSO = 4:1 (red) as solvent

To understand the unique spinnability of PAAS solutions in water/DMSO mixture solvent, rheological measurements were conducted to study the viscoelastic properties of PAAS solutions. As shown in Supplementary Fig.8, the storage moduli (*G’*) are dominant over loss moduli (*G”*) across the whole range of frequencies studied, which indicates the viscoelastic behavior of PAAS solutions. The optimal PAAS solution (4 wt%) appeared as a physical gel at room temperature (Fig. 2a), and the high viscosity of the gel is an important factor for drawing good PAH fibers. At lower concentrations (e.g., 2 wt%), the PAAS solution remained as viscous liquid and the filaments drawn from this solution were thinner and unstable (Supplementary Fig. 9). At higher concentrations (e.g., 6 wt%), PAAS precipitated out of the solution upon cooling to room temperature. Strain-dependent oscillatory rheology of the optimal PAAS solution displays a broad linear gel region with a gel to sol cross-over point appearing at 447% strain (Fig. 2b). In the step-strain measurements (Fig. 2c), the optimal PAAS solution exhibits a fast and complete recovery to its initial modulus, which indicates a great reversibility due to the fast alignment and relaxation of PAAS chains in the physical gel^37^. As shown in Fig. 2d, both *G’* and *G”* values of a 4 wt% PAAS solution in pure water are higher than that of the optimal PAAS solution (4 wt% PAAS in water/DMSO solvent), indicating that PAAS chains are more extended in pure water than in water/DMSO mixture. However, filaments drawn from this 4 wt% PAAS solution in pure water were thinner and unstable, which could not form PAH fibers (Supplementary Fig. 10). Reduction of the content of DMSO (e.g., from 20 wt% to 14.3 wt%) would also result in thinner and fragile PAH fibers (Supplementary Fig. 11). These results clearly demonstrate the key effect of DMSO, which triggers the quick phase separation to form PAH fibers.

Due to the polyelectrolyte nature of PAAS, pH and salts are also expected to affect the behavior of PAAS solutions. The optimal PAAS solution is weakly basic (pH ~ 8), where the majority of carboxylate groups are negatively charged. In an acidic solution, PAAS chain is protonated and the uncharged linear polymer chain prefers a random coil conformation in solution^37^. Therefore, when adjusting the PAAS solution to acidic pH (e.g., from 8 to 1), the solution viscosity was greatly reduced and no PAH fibers can be obtained (Supplementary Fig. 12). On the other hand, adding salts into the PAAS solution could benefit the formation of PAH fibers. Using NaCl as one example, increasing NaCl concentration from 0 to 50 mM can significantly accelerate the gelation rate of PAAS filament in air, leading to the formation of thicker PAH fibers (Supplementary Fig. 13). The effect of NaCl was observed by using rheological measurements (Supplementary Fig. 14): the gel to sol cross-over point was increased from 447% to 680% strain simply by adding 50 mM NaCl into the optimal PAAS solution, which indicates the enhanced polymer–polymer interactions by addition of NaCl into solution^37^.

### Mechanical performance of PAH and MAPAH fibers

**Fig. 3:**
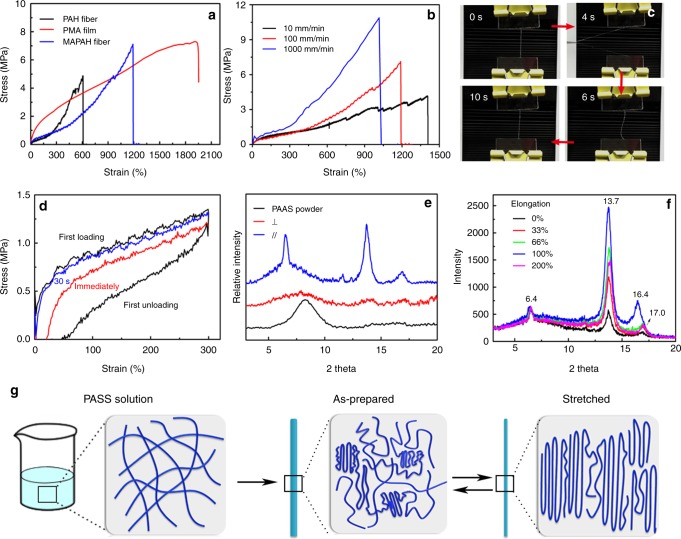
Mechanical and structural characterization of hydrogel fibers. **a** Stress–strain profiles of a PMA film, PAH, and MAPAH fibers at 100 mm min^−1^ stretching rate. **b** Stress–strain profiles of a MAPAH fiber under different stretching rates. **c** Photograph of a MAPAH fiber showing the hysteresis effect during its recovery process. **d** Stress–strain profiles of a MAPAH fiber subjected to a loading–unloading cycle at 300% strain (black curve). Immediately after the 1st cycle, the fiber was stretched to 300% strain (red curve). The same fiber was unloaded and stretched to 300% strain after a 30 s recovery at ambient condition (blue curve). **e** XRD spectra of PAAS powder, and MAPAH fibers placed parallel or perpendicular to the X-ray incidence direction. **f** XRD spectra of MAPAH fibers under different stretching strains. **g** Proposed molecular organization and orientation of PAAS chains at different conditions. PAAS is solvated and randomly oriented in solution. Coexistence of crystalline and amorphous domains in as-prepared hydrogel fibers. Mechanical stress enables PAAS chains a higher degree of crystallization and orientation along the strain direction

The mechanical properties of both PAH and MAPAH fibers were studied based on fibers drawn from the same optimal PAAS solution, whose diameters were controlled as 200 ± 20 μm by adjusting the drawn speed in a proper range. The environmental temperature and relative humidity were maintained at 25 °C and 42 ± 2%, respectively. The water content of PAH fibers and MAPAH fibers measured by thermogravimetric analysis (TGA) were similar, both of which were in the range of 25 ± 3% (Supplementary Fig. 15). Representative stress–strain curves of PAH and MAPAH fibers show a rubber-like characteristics (Fig. 3a)^38^, indicating the viscoelastic nature of both fibers. Remarkably, due to the good balance of strength (4.4 ± 0.5 MPa) and stretchability (elongation at break of 740 ± 100%), PAH fibers achieved a high tensile toughness of 11.1 ± 0.5 MJ m^−3^, which is the total energy required to break the fiber. More surprisingly, greatly enhanced tensile strength (5.6 ± 0.6 MPa) and stretchability (elongation at break of 1180 ± 100%) were simultaneously achieved by MAPAH fibers, resulting in a tensile toughness of 26.8 ± 3.1 MJ m^−3^ that is among the best of current tough hydrogels^11,39–41^. Since the PMA film has a much higher stretchability (elongation-at-break ~1900%) than the PAH fiber, the very thin PMA coating layer (thickness 2–3 μm) may reinforce the PAH fiber (diameters of 200 ± 20 μm) by reducing the generation of crack on the fiber surface. Therefore, the mechanical properties of the MAPAH fiber is significantly better than the PAH fiber. Due to the great water-resistance and enhanced mechanical properties of MAPAH fibers, we have focused onto MAPAH fibers for further exploration.

Spider silk shows strain-rate dependent mechanical properties^28^ and hysteresis behavior upon deformation and recovery^41^. Similar to spider silk, the mechanical performance of MAPAH fibers is also strain-rate dependent: the measurement at a higher stretching rate gives a higher tensile strength and a lower stretchability (Fig. 3b). The measured tensile toughness of MAPAH fiber increases with the increase of stretching rate, from 29.2 MJ m^−3^ at 10 mm min^−1^ to 30.9 MJ m^−3^ at 100 mm min^−1^, and further to 45.0 MJ m^−3^ at 1000 mm min^−1^, which implies that MAPAH fibers are even tougher upon fast impact or collision. As another feature similar to spider silk, MAPAH fibers show a clear hysteresis behavior during its recovery from large deformation (Supplementary Movie 3). Firstly, a MAPAH fiber was kept straight and tight between two clamps. After being stretched and released, the fiber was loose at the very beginning and gradually returned to the straight and tight status within 5 s (Fig. 3c). The recovery process was further studied by a cyclic loading test. A MAPAH fiber was stretched to 300% strain and unloaded. If the fiber was reloaded immediately after unloading, the reloading curve indicated a moderately weakened fiber in comparison with the initial curve. Amazingly, when the fiber was reloaded 30 s after the last unloading, the reloading curve almost coincided with the initial one, which indicates a complete recovery of the hydrogel fiber within 30 s at room temperature (Fig. 3d). Upon stretching, previous tough hydrogels with sacrificial non-covalent or dynamic covalent bonds can dissipate energy efficiently by breaking these bonds. But the recovery of these tough hydrogels is relatively slow (from several hours to several days), due to the slow chain motion in the crosslinked hydrogel matrix^11,39,40^. In contrast, MAPAH fibers show both high tensile toughness and fast recovery in several seconds, which implies a mechanism of dissipating energy and recovery that is different from previous tough hydrogels.

### Structural analysis of MAPAH fibers

To understand the mechanism of the unique mechanical behavior of MAPAH fibers, we sought to study the chemical composition and macromolecular alignment of MAPAH fibers by using Infrared Spectroscopy (IR) and X-ray diffraction (XRD), respectively. As shown in Supplementary Figs.1, 6, the IR spectrum of PAH is identical with that of PAAS, while the IR spectrum of MAPAH is just an arithmetic sum of the spectrum of PAAS and PMA, indicating no significant change on chemical bonding structure during the preparation of PAH and MAPAH fibers. On the other hand, the XRD spectrum of PAAS powder and PMA film shows only one broad peak, indicating their amorphous nature (Fig. 3e, Supplementary Fig. 17). In contrast, the XRD spectrum of both PAH and MAPAH fibers clearly shows three sharp peaks at 2*θ* = 6.4°, 13.7°, 17.0° (Supplementary Fig. 17), which indicates the presence of crystalline domains in both PAH and MAPAH fibers. Meanwhile, these diffraction peaks are the strongest when the fiber’s length direction and the X-ray incidence direction are parallel to each other, and disappear when the two directions are perpendicular to each other, which demonstrates that the orientation of PAAS chains is along the length direction of fibers. Based on molecular mechanics simulation (Supplementary Fig. 18, see SI for details), PAAS chains in a crystalline bundle prefer an α-helix-like conformation that has a pitch around 6.5Å, corresponding to the diffraction peak at 13.7 °. The average distance between α-helix is around 5.4Å, corresponding to the peak at 17.0°. When a free MAPAH fiber was stretched, the relative intensity of the peak at 13.7 ° can be increased up to 5 times (Fig. 3f), indicating the presence of amorphous domain in MAPAH fibers that can be induced by mechanical stress to form crystalline domains.

## Discussion

Based on the structural analysis, a mechanism was proposed (Fig. 3g) to explain the unique mechanical behavior of MAPAH fibers. In the PAAS solution, PAAS chains are expanded and randomly distributed. Upon drawn from the gel-like solution, orientation and crystallization of PAAS chains proceed effectively along the fiber direction to form crystalline domains as physical crosslinking points. These crystalline domains are connected by soft amorphous domains, which could be similar to the structure of spider dragline silk^26^. Since MAPAH fibers contain a large amount of water that serve as solvent and plasticizer, the amorphous domains could be forced to form ordered alignment upon stretching, providing a high stretchability and high strength. After the stretched fiber being released, the temporarily aligned PAAS chains would relax and adopt entropically favorable random conformation and orientation, enabling the fast recovery with the assistance of water in fibers. Therefore, the high stretchability and fast resilience of MAPAH fibers can be attributed to the stress-driven alignment and entropy-driven molecular relaxation of PAAS chains mainly in the amorphous domains, while the crystalline domains serve as physical crosslinking points and enable a high tensile strength^21^.

**Fig. 4:**
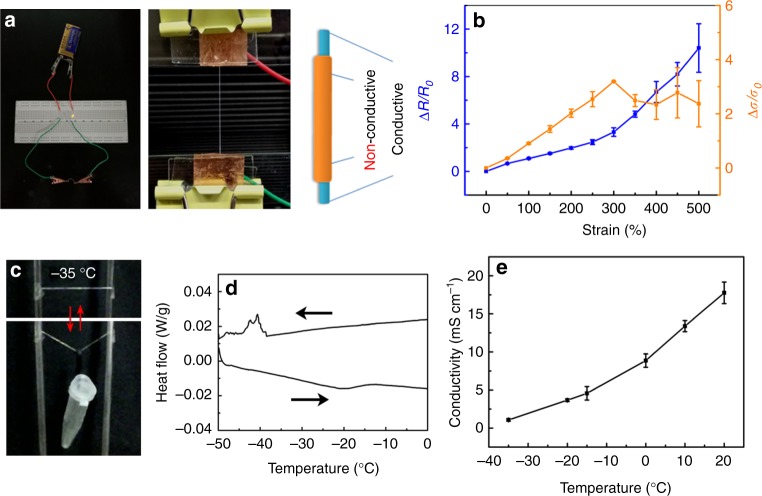
Electrical conductivity of stretchable and anti-freezing MAPAH fibers. **a** MAPAH fibers can serve as a highly stretchable wire with the PAAS hydrogel as a conductive core and the PMA coating layer as an insulating cover. **b** Relative resistance variation Δ*R*/*R*_0_ and relative conductivity variation Δ*σ*/*σ*_0_ of a MAPAH fiber upon stretching at 25 °C. **c** A MAPAH fiber under repeated weight-bearing test at −35 °C with a plastic vial ~800 times heavier than the fiber. **d** DSC of MAPAH fibers shows a phase transition at around −40 °C. **e** Conductivity of MAPAH fibers in the temperature range of −35–25 °C. The error bar for each data point in **b** and **e** is standard deviation calculated based on 6–8 parallel measurements

Besides the outstanding mechanical properties, a MAPAH fiber is electrically conductive due to the polyelectrolyte nature of PAAS. Indeed, a MAPAH fiber can serve as a highly stretchable wire in a circuit (Fig. 4a), with a conductive PAAS hydrogel core and an insulating PMA cover. The MAPAH fibers can be repeatedly stretched, resulting in a periodical change of light intensity of the LED in this circuit (Supplementary Movie 4). The conductivity of MAPAH fibers was measured as ~2 S m^−1^ at room temperature, which is in the conductivity range of concentrated PAAS solutions in water (1–4 S m^−1^)^29^. As shown in Supplementary Fig. 1,9, the PMA coating has a very good insulation property. To probe the conductive performance of MAPAH fibers upon stretching, the electrical resistance variation ratio (Δ*R*/*R*_0_ = (*R*−*R*_0_)/*R*_0_; *R*_0_ and *R* correspond to the resistance without and with stretching, respectively) as a function of the strain was studied (Fig. 4a). From 0 to 300% elongation, the electrical resistance of MAPAH fibers increases almost linearly with the strain (Fig. 4b). Further stretching to 500% strain leads to a faster increase of resistance with a bigger fluctuation. Even stretched to 1000% strain, the MAPAH fiber remains conductive (Supplementary Fig. 20). Interestingly, the calculated conductivity of MAPAH fibers also increases upon stretching. The highest conductivity was achieved at 300% strain, which was ~4 times of the initial conductivity of MAPAH fibers. Since the strain-dependent change of electrical resistance is fully reversible, the large increase of conductivity upon stretching could be attributed to the enhanced orientation of PAAS chains along the fiber length direction, which may facilitate the diffusion of sodium ions along the PAAS chains^29^.

Conventional conductive hydrogels would freeze and lose stretchability and conductivity at subzero temperatures. Liu has reported organohydrogels based on water-ethylene glycol binary solution that can sustain subzero temperature due to the anti-freezing property of water-ethylene glycol mixture^23^. Repeated weight-bearing test of MAPAH fibers at low temperature demonstrates that the fiber retains its great stretchability and fast resilience even at −35 °C (Fig. 4c). Indeed, the differential scanning calorimetric (DSC) measurement of MAPAH fibers shows a phase transition around −40 °C (Fig. 4d), which is likely corresponding to the freezing point of water in MAPAH fibers. The DSC result and the great stretchability at low temperatures indicate that MAPAH fibers possess a remarkable anti-freezing property. Upon the decrease of temperature, the conductivity of MAPAH fibers decreases due to the reduction of ion diffusion rate, and remains 0.1 S m^−1^ at −35 °C (Fig. 4e). The molar ratio of H_2_O:DMSO in this fiber was measured by using ^1^H NMR as ~1300:1 (Supplementary Fig. 21), which indicates water is the only solvent in the MAPAH fiber. The absence of anti-freezing organic solvents in MAPAH fiber implies a different anti-freezing mechanism from organohydrogels. Indeed, the outstanding anti-freezing property of MAPAH fibers is likely due to the high concentration of sodium ions in the PAAS hydrogel matrix, since concentrated salt solution is anti-freezing. The conductivity of MAPAH fibers is comparable to other conductive hydrogels at room temperature^13–16^ and much higher than that of organohydrogels at low temperatures^23^, which indicates the advantage of MAPAH fibers as stretchable and anti-freezing conductive hydrogel fibers.

In summary, we report ultra-stretchable and anti-freezing conductive hydrogel fibers that are facilely prepared by spinning of PAAS solutions at ambient conditions. Crystalline and amorphous domains are coexistent in the as-prepared hydrogel fibers, which may reversibly convert between each other upon mechanical stress and release. The dynamic alignment and relaxation of PAAS chains in the amorphous domains are likely responsible for the large stretchability and fast resilience, while the crystalline domains could serve as physical crosslinking points and enable the high tensile strength. MAPAH fiber can serve as a highly stretchable and anti-freezing wire with good electrical conductivity. Owing to the ordered and reversible chain alignment enabled by the spinning and gelation process, MAPAH fiber represents a unique example of high-performance and low-cost stretchable conductive fibers, which will shed light on the design of next generation textile-based stretchable electronic devices.

## Methods

### Materials

Sodium polyacrylate (PAAS, *M*_w_ ~ 3 × 10^7^ Da) was purchased from Sinopharm Chemical Reagent Co. Ltd. Methyl acrylate (MA) was passed through a basic alumina column to remove the inhibitor before polymerization. Tetrahydrofuran (THF) was dried over molecular sieves. 2,2’-Azobisisobutyronitrile (AIBN) was recrystallized twice from ethanol. All other chemicals were of analytical grade, purchased from Sinopharm Chemical Reagent Co. Ltd. and used as received.

### Fabrication of PAH fibers and MAPAH fibers

The optimal PAAS solution was prepared as follows: 200 mg PAAS solid powder in 4.8 g solvent (3.84 g H_2_O and 0.96 g DMSO) was stirred and heated at 80 °C for 1 h to get a uniform, transparent and viscous solution. As shown in Supplementary Movie 1, PAH fibers were directly drawn out of the corresponding PAAS solutions at room temperature. And it needs 1–2 min to let the fiber solidify in room temperature air. The formed PAH fibers were immersed in a 5% PMA/ethyl acetate solution for 5–10 s, and then taken out to let the ethyl acetate solvent evaporate. A very thin layer of PMA was coated on the PAH fibers to form the core–shell MAPAH fibers. The PAH and MAPAH fibers for mechanical tests were drawn from the same optimal PAAS solution (4 wt% PAAS in water/DMSO mixture with 20 wt% DMSO), whose diameters were controlled as 200 ± 20 μm by adjusting the drawn speed.

### Characterization of PAH fibers and MAPAH fibers

Field emission scanning electron microscope (FE-SEM, JEOL JSM-6700F) was used to image the freeze-dried hydrogel fibers. For all the other characterization, freshly prepared hydrogel fibers were used. X-ray diffraction (XRD) was carried out on a Rigaku D X-ray diffractometer with Cu Kα radiation (*λ* = 1.54178 Å). One MAPAH fiber was placed parallel or perpendicular to the X-ray incidence direction. For the XRD of stretched MAPAH fibers, one MAPAH fiber with certain elongation was glued on a silicon wafer and placed parallel to the X-ray incidence direction. Thermogravimetric analysis (TGA) was done by TGA Q5000IR, the temperature was from 20 °C to 800 °C with 10 °C/min. Differential scanning calorimetric (DSC) was performed by using TA Q2000 at identical heating and cooling rate of 2 °C min^−1^ between −50 °C and 90 °C. The contact angle of water droplet on PMA film and MAPAH fiber was measured by using a contact angle meter SL2008, Solon Tech. Rheological measurements of different PAAS solutions were conducted on a TA AR-G2 rheometer. Mechanical properties of PAH fibers and MAPAH fibers were tested on an Instron 3340 universal testing instrument at 25 °C and relative humidity ~42 ± 2%. The average diameter of each fiber sample was obtained from three measurements along the fiber length using an optical microscope. The electrical resistance of MAPAH fibers was measured by using a multimeter. The electric conductivity was calculated based on the equation: *σ* = *L*/(*S* × *R*), where *L*, *S*, *σ*, and *R* are the length, cross section area, conductivity, and electrical resistance of the MAPAH fiber.

## Electronic supplementary material

Supplementary Information

Description of Additional Supplementary Files

Supplementary Movie 1

Supplementary Movie 2

Supplementary Movie 3

Supplementary Movie 4

## Data Availability

The authors declare that the data supporting the findings of this study are available within the article and its Supplementary Information.
