# Supplementary material for: Bioinspired ultra-stretchable and anti-freezing conductive hydrogel fibers with ordered and reversible polymer chain alignment
Source: Nat Commun. 2018 Sep 4;9:3579. doi: 10.1038/s41467-018-05904-z (PMC6123392; doi:10.1038/s41467-018-05904-z)
Supplement: Supplementary file 1 — Supplementary Information [file 41467_2018_5904_MOESM1_ESM.pdf]

Supporting Information

Bioinspired Ultra-stretchable and Anti-freezing Conductive Hydrogel Fibers with Ordered and Reversible Polymer Chain Alignment

Zhao et al

Supplementary Methods

Synthesis and characterization of polymethyl acrylate (PMA)

In a typical synthesis, MA (3.44 g, 40 mmol), dried THF (6 mL) and AIBN (2.1 mg, 13 μmol) were added to a Schlenk tube. The tube was purged by nitrogen to remove oxygen, and then placed into an oil bath preheated to 60 °C. The solution was stirred by a magnetic stirred at 200 rpm. The polymerization was done after 24 h. The product was precipitated out by adding the solution into 100 mL ethanol, washed with ethanol, and dried under vacuum. The PMA yield was typically 80%, which was characterized by gel permeation chromatography (GPC) analysis. GPC analysis: PMA, $M_n = 3.8\times10^5$ Da, PDI = 1.93. PMA was dissolved in ethyl acetate to give a 5 wt% PMA solution.

Preparation of concentrated PAAS solutions:

(1) PAAS solutions with different concentrations were prepared as follows. Taking 4% PAAS solution as one example, 200 mg PAAS solid powder in 4.8 g solvent (3.84 g H_2O and 0.96 g DMSO) was stirred and heated at 80 °C for 1h to get a uniform, transparent and viscous solution. PAAS solutions of different concentrations were prepared by varying the amount of PAAS.

(2) 4% PAAS solutions with different solvents were prepared as follows: 200 mg PAAS was dissolved in 4.8 g solvent by stirring and heating at 80 °C for 1 h to get a uniform, transparent and viscous solution. The solvent was prepared by mixing H_2O and DMSO at different weight ratio, H_2O : DMSO = 2:1, 3:1, 4:1, 5:1, 6:1.

(3) 4% PAAS solutions (H_2O : DMSO = 4 : 1) with different NaCl concentrations. NaCl was dissolved in H_2O to obtain 25, 50 and 100 mM NaCl solution. Then 3.84 g NaCl solution and 0.96 g DMSO was mixed to dissolve 200 mg PAAS at 80 °C for 1 h to get a uniform, transparent and viscous solution.

(4) Acidic 4% PAAS solution. 3.84 g 1.0 M HCl solution and 0.96 g DMSO was mixed to dissolve 200 mg PAAS at 80 °C for 1h to get a uniform, transparent and viscous solution. The final pH was ~ 1. These as-prepared PAAS solutions were allowed to cool down to room temperature before use.

Fabrication of artificial webs based on PAH fibers or MAPAH fibers: An artificial web was weaved by using PAH fibers on a circular frame. The as-prepared PAH fibers were sticky and can

form good adherence to the frame and to each other. After a web based on PAH fibers was obtained, the web was gently immersed into PMA/ethyl acetate solution to coat a thin layer of PMA on the web to form a waterproof MAPAH web.

Characterization methods: Field emission scanning electron microscope (FE-SEM, JEOL JSM-6700F) was used to image the freeze-dried hydrogel fibers. For all the other characterization, freshly prepared hydrogel fibers were used. X-ray diffraction (XRD) was carried out on a Rigaku D X-ray diffractometer with Cu Kα radiation (λ=1.54178 Å). One MAPAH fiber was placed parallel or perpendicular to the X-ray incidence direction. For the XRD of stretched MAPAH fibers, one MAPAH fiber with certain elongation was glued on a silicon wafer and placed parallel to the X-ray incidence direction. Thermogravimetric Analysis (TGA) was tested by TGA Q5000IR, the temperature was from 20 °C to 800 °C with 10 °C min^{-1}. Differential Scanning Calorimetric (DSC) was performed by using TA Q2000 at identical heating and cooling rate of 2 °C min^{-1} between -50 °C and 90 °C. The DSC measurement was firstly cooled from 90 °C to -50 °C, and then heated from -50 °C to 90 °C. The DSC sample was kept under inert environment using continuous nitrogen gas purging at 50 ml min^{-1} flow rate. Gel permeation chromatography was performed by using a Shimadzu Prominence GPC with HPLC grade THF as solvent. A thin PMA layer was spin-coated on a flat glass slide for contact angle measurement. The contact angle of water droplet on PMA film and MAPAH fiber was performed by using a contact angle meter SL2008, Solon Tech. Co.,Ltd.

Rheological studies: Rheological measurements were conducted on a TA AR-G2 rheometer using a cone-plate of 40 mm diameter with a cone angle of 1°. The frequency-sweep spectra were recorded in a constant-strain (0.1%) mode over the frequency range of 0.1–100 rad s^{-1} at 25 °C. The strain-sweep spectra were recorded in a constant frequency of 10 rad s^{-1} over the strain range of 1-1000% at 25 °C. Step-strain measurement was performed with applied oscillatory strain alternated between 1 and 1000% for 30 s periods (ω = 10 rad s^{-1}, 25 °C). The PAAS solutions at 80°C was added into the holder of rheometer and cooled down to room temperature before test.

Water-resistance test of PAH and MAPAH fibers: As shown in Movie S2, a PAH or MAPAH fiber was hold straight between two clamps. Liquid water droplet was put in contact with the fibers.

As shown in Movie S3, a PAH or MAPAH web was hold on a frame. Liquid water droplet was put in contact with the webs. In both cases, PAH fiber was broken very quickly after contacting liquid water, while MAPAH fiber remained stable and stretchable after contacting liquid water.

Mechanical test: Mechanical properties of PAH fibers and MAPAH fibers was tested on an Instron 3340 universal testing instrument at 25 °C and relative humidity ~ 42±2%. The average diameter of each fiber sample was obtained from three measurements along the fiber length using an optical microscope. The fiber's diameter was in the range of 180-220 μm. The initial distance between the two clamps on the Instron machine was 10 mm. The elongation rate was at 100 mm min^{-1}, unless otherwise stated.

To probe the mechanical performance of MAPAH fibers at low temperatures, a MAPAH fiber was hold between a two-arm metal holder (as shown in Figure 4c) and put into a freezer (-35 °C). The fiber was equilibrated at -35 °C for >30 min. Then, a plastic vial of ~ 2 gram was hold onto the fiber, which caused elongation of the fiber. The weight of the vial was about 800 time of the weight of MAPAH fiber. After removal of the vial, the fiber quickly returned to its initial status. The stretching and resilience of MAPAH fiber can be repeated many times at -35 °C, indicating a good anti-freezing property of MAPAH fiber.

Measurement of MAPAH fiber's conductivity at different conditions: The electrical resistance of MAPAH fibers was measured by using a multimeter. The average diameter of each fiber sample was obtained from three measurements along the fiber length using an optical microscope. The electric conductivity was calculated based on the equation: $\sigma = L/(S\cdot R)$, where L, S , σ and R are the length, cross section area, conductivity and electrical resistance of the MAPAH fiber.

The measurement of electrical resistance of MAPAH fibers with different elongation strain was performed at 25 °C and relative humidity ~ 42±2%. A MAPAH fiber was hold between two clamps. Both ends of the fiber was connected to a copper sheet for the measurement of electrical resistance during stretching. At each strain, the cross section area of the fiber was calculated by assuming the total volume of the stretched fiber remains constant. This assumption has been tested by measuring the diameter of stretched MAPAH fibers under an optical microscope. The measured diameter and the predicted diameter are very close to each other.

The measurement of conductivity of MAPAH fibers at low temperatures was performed by putting the fiber in a freezer with adjustable temperature from -35 °C to 0 °C. The MAPAH fibers were equilibrated at a certain temperature for >30 min before the measurement of electric resistance. The change of length and diameter of MAPAH fiber upon the temperature change in this range was negligible.

Measurement of the DMSO content in a MAPAH fiber by ^{1}HNMR:

To measure the DMSO content in the MAPAH fiber, a known weight of MAPAH fiber (~ 20 mg) was gradually dissolved in hot D_2O and measured by ^{1}H NMR. A certain amount of dichloroacetic acid (DCA) was added to D_2O as an internal reference. Before addition of the MAPAH fiber, the area of water peak in D_2O was measured as w_1. After dissolved the fiber, the area of water peak and DMSO peak was measured as w_2 and D. Therefore, the molar ratio of H_2O : DMSO in the MAPAH fiber can be calculated as H2O : DMSO = $[(w_2-w_1)/2] : (D/6)$. Based on the data shown in Supplementary Figure 20, the molar ratio of H_2O : DMSO is calculated as 1318.5 :1, which means that the DMSO content in the MAPAH fiber is very low. The PAAS polymer shows no observable peak in the ^{1}H NMR due to its ultrahigh molecular weight. The PMA coating was not soluble in D_2O. The low content of DMSO in the MAPAH fiber could be because that most of DMSO in the PAH fiber has been extracted out by ethyl acetate solvent during the PMA coating process.

Molecular mechanics simulation of the alignment structure of PAAS chains:

Molecular mechanics simulation was performed using Gaussian 16 [1] on the supercomputing system in the Supercomputing Center of University of Science and Technology of China. Universal Force Field (UFF)[2] and Polarizable continuum model (PCM)[3] were used for the optimization. Three PAAS chains (7 acrylic acid units on each chain) were used for the simulation. In the optimized structure (Supplementary Figure 15), the three PAAS chains are roughly parallel to each other. For each PAAS chain, it takes a α-helix-like conformation. In each turn of the helix, there are four acrylic acid units. The pitch of the helix is ~ 6.5 Å, while the average distance between two helix is ~ 5.4 Å.

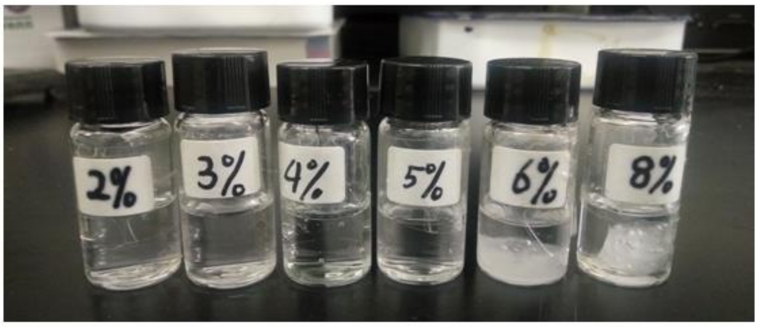

Supplementary Figure 1. PAAS solutions of different concentrations in H_2O:DMSO = 4:1 solvent**.** For 6% and 8% solutions, PAAS was completely dissolved at 80 ^{o}C and precipitated out after cooling to room temperature.

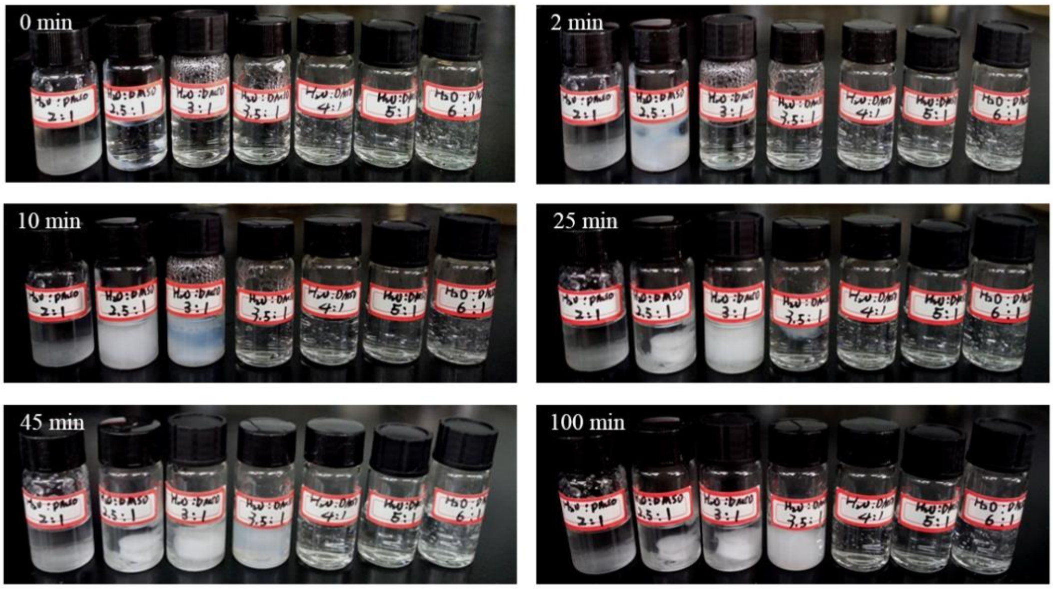

Supplementary Figure 2. The phase separation process of 4% PAAS solutions in H_2O/DMSO mixture with different H_2O:DMSO ratio. 4% PAAS was completely dissolved at 80 ^{o}C in H_2O/DMSO mixture, except for the one with H_2O:DMSO = 2:1. At room temperature, the PAAS solutions slowly cooled down and phase separation was observed by the formation of white precipitates, which started earlier with higher content DMSO in the solution. When the PAAS solutions cooled down to room temperature, the solution with H_2O:DMSO = 3.5:1 showed phase separation, while the solution with H_2O:DMSO = 4:1 remained transparent, indicating the critical concentration of DMSO is between 20% and 22.2%.

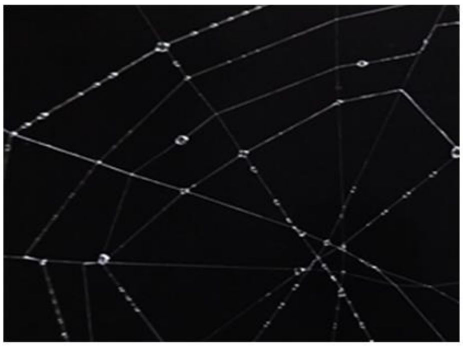

Supplementary Figure 3. The PAH web shows the beads-on-a-string structure, similar to the spider web.

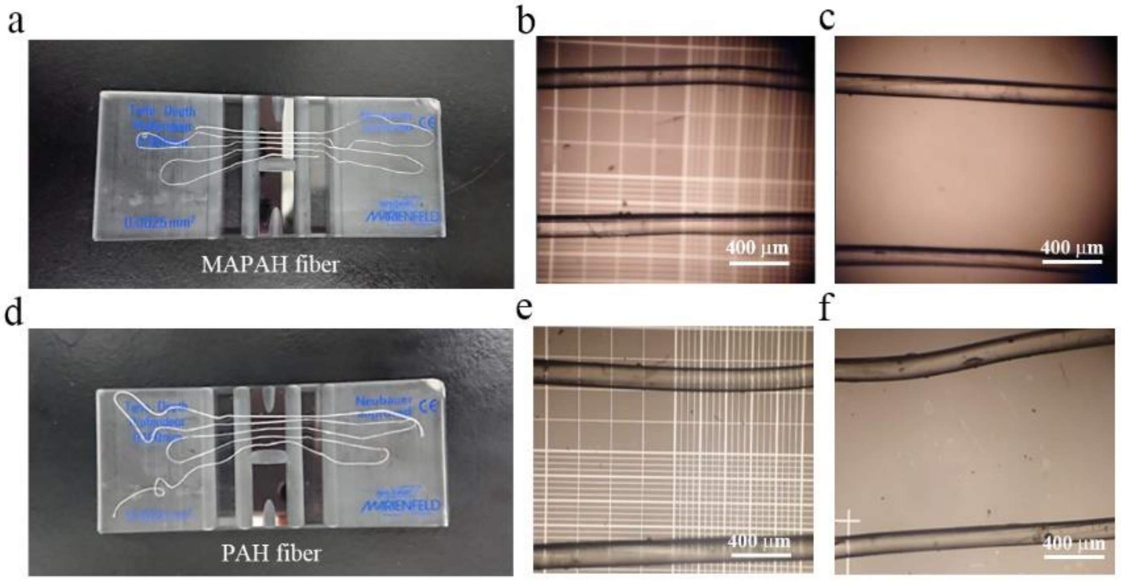

Supplementary Figure 4. Photographs of MAPAH and PAH fibers. a-c) images of a MAPAH fiber; d-f) images of a PAH fiber.

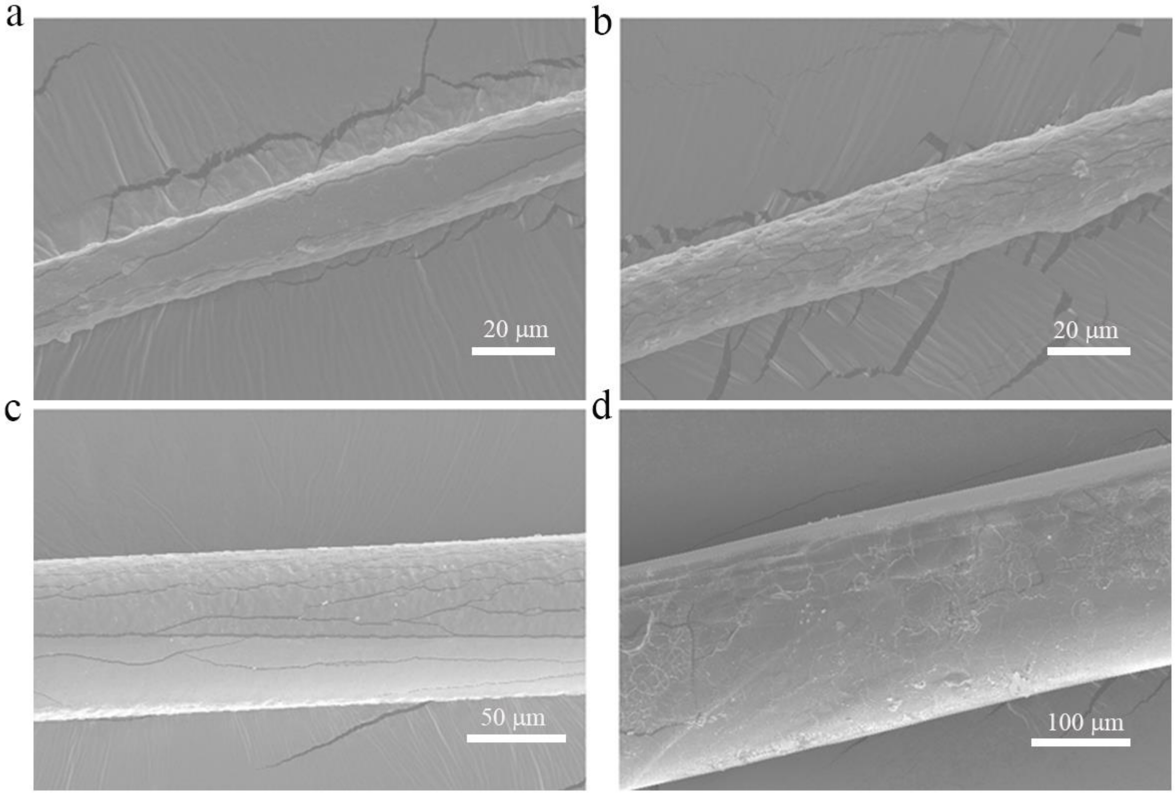

Supplementary Figure 5. SEM images of PAH fibers with different diameters. The cracks on the surface of PAH fibers was caused by the freeze-drying process.

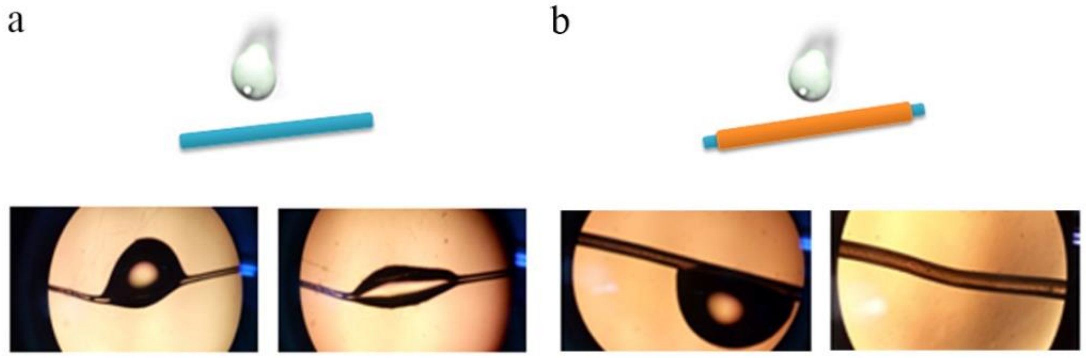

Supplementary Figure 6. The hydrophilic property of PAH fibers and MAPAH fibers observed under optical microscope. a) The PAH fiber is swelled and gradually dissolved by a water droplet. b) The MAPAH fiber does not swell by water droplet. After the water droplet was absorbed by a tissue paper, the MAPAH fiber remains unchanged.

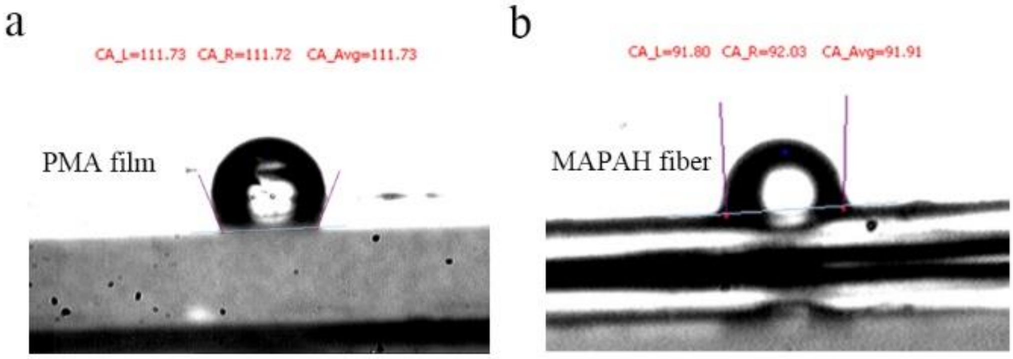

Supplementary Figure 7. Measurement of the contact angle of a PMA film and a MAPAH fiber. a) Image of a water droplet on a flat PMA layer coating on glass. b) Image of a water droplet on a MAPAH fiber.

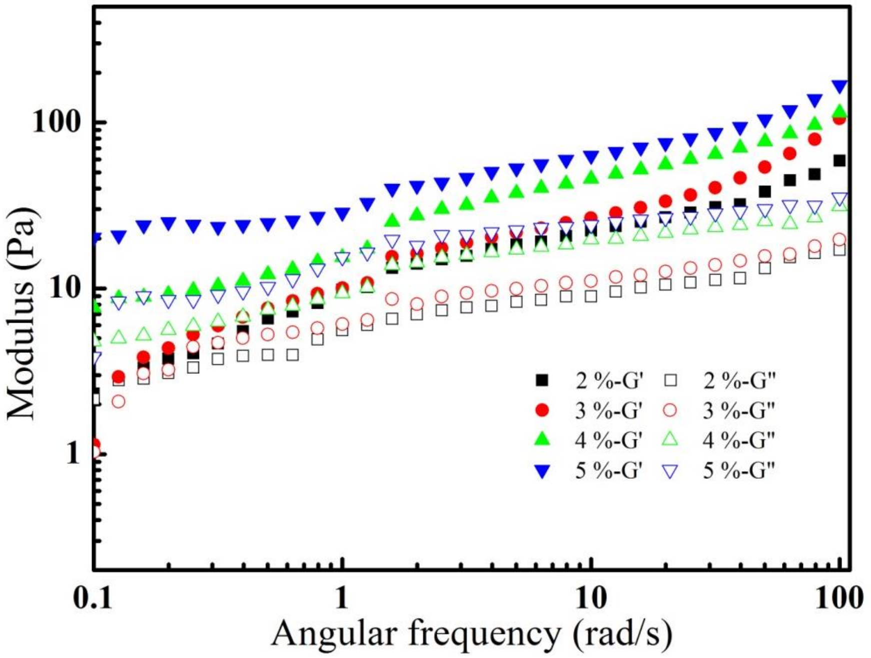

Supplementary Figure 8. Rheological characterization of PAAS solutions in the H_2O:DMSO = 4:1 solvent with different PAAS concentration, from 2% to 5%.

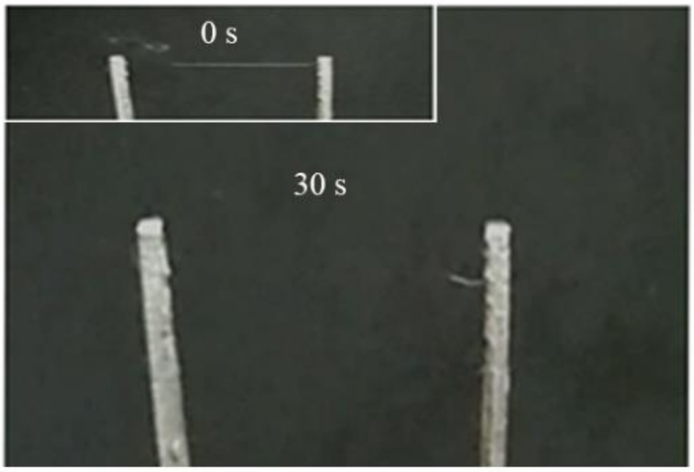

Supplementary Figure 9. A viscous filament drawn from a 2% PAAS solution in H_2O:DMSO = 4:1 mixture was not stable, which was broken within 30 s.

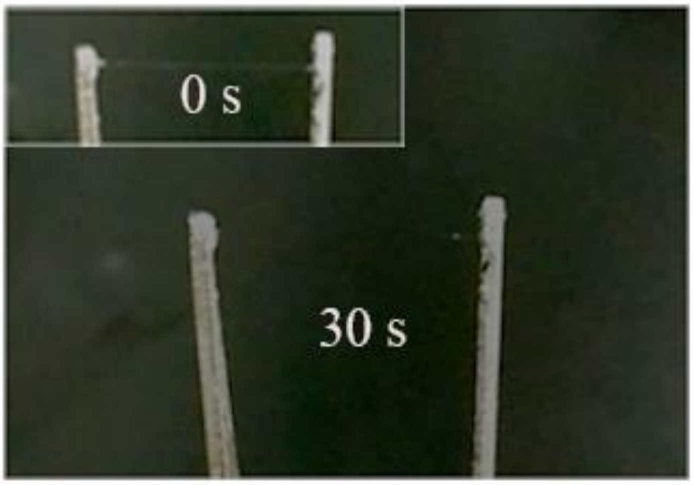

Supplementary Figure 10. A viscous filament drawn from a 4% PAAS solution in pure water was very thin and not stable, which was broken within 30 s.

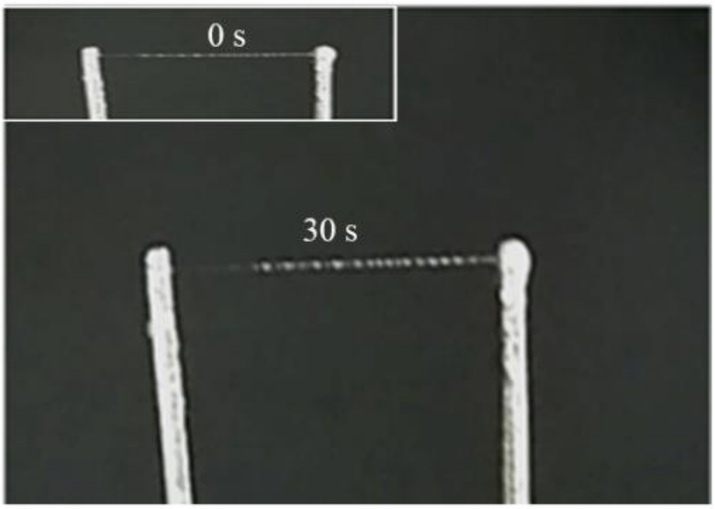

Supplementary Figure 11. A viscous filament drawn from a 4% PAAS solution in H_2O:DMSO = 6:1 mixture was very thin and fragile.

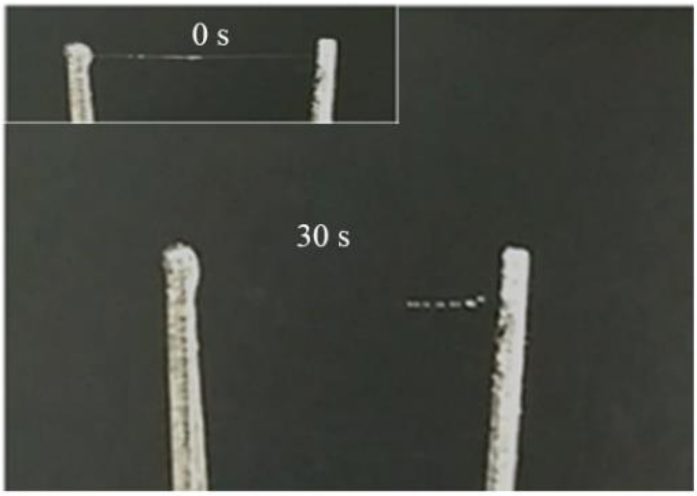

Supplementary Figure 12. A viscous filament drawn from a 4% PAAS acidic solution in H_2O:DMSO = 4:1 mixture solution (pH ~ 1) was very thin and fragile.

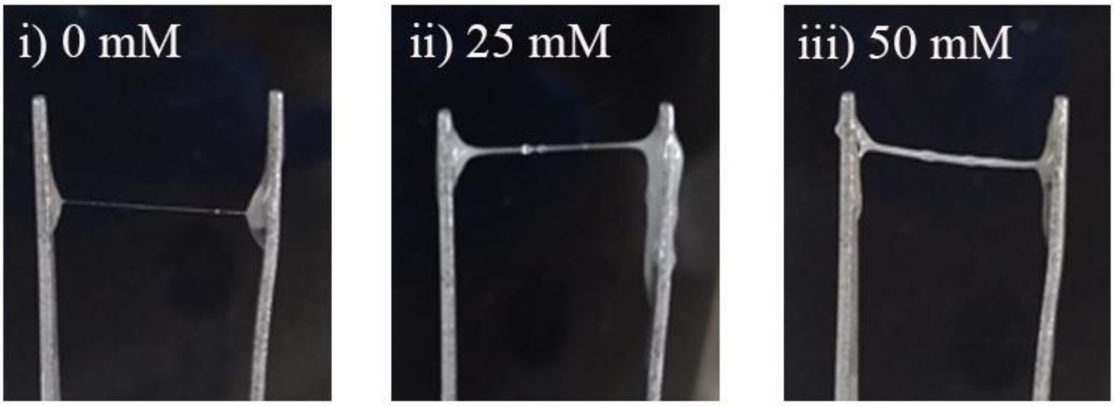

Supplementary Figure 13. A viscous filament drawn from a 4% PAAS solution in H_2O:DMSO = 4:1 mixture solution with different concentration of NaCl (0, 25, 50 mM NaCl).

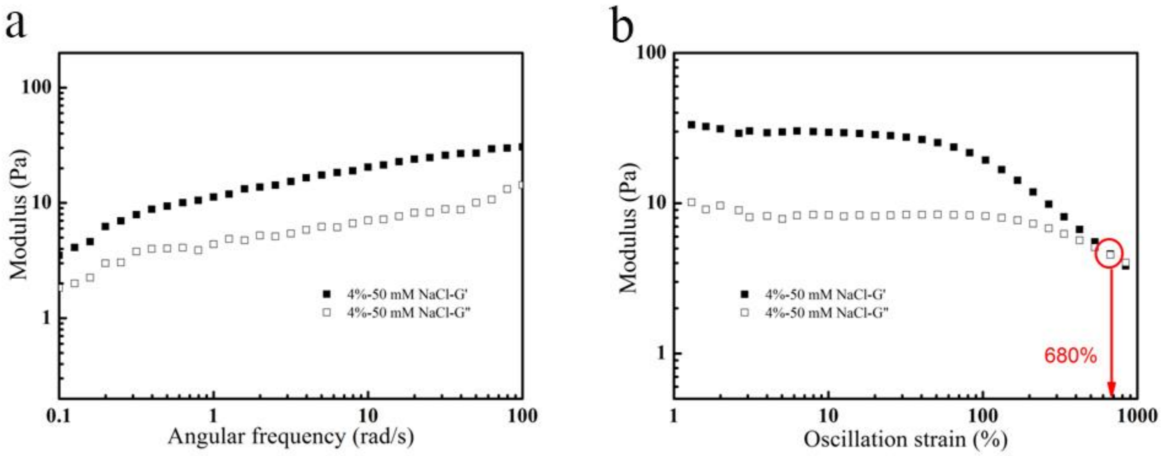

Supplementary Figure 14. Rheological characterization of 4% PAAS solution in the H_2O:DMSO = 4:1 solvent with 50 mM NaCl. a) Frequency dependent oscillatory rheology of this solution. The storage modulus (*G'*) is larger than the loss modulus (*G''*) indicating the gel behavior. b) Rheological strain oscillatory rheology of this solution from 1 to 1000% at 25 °C (ω= 10 rad s^{-1}). The *G'*/*G''* cross-over point was at 680%.

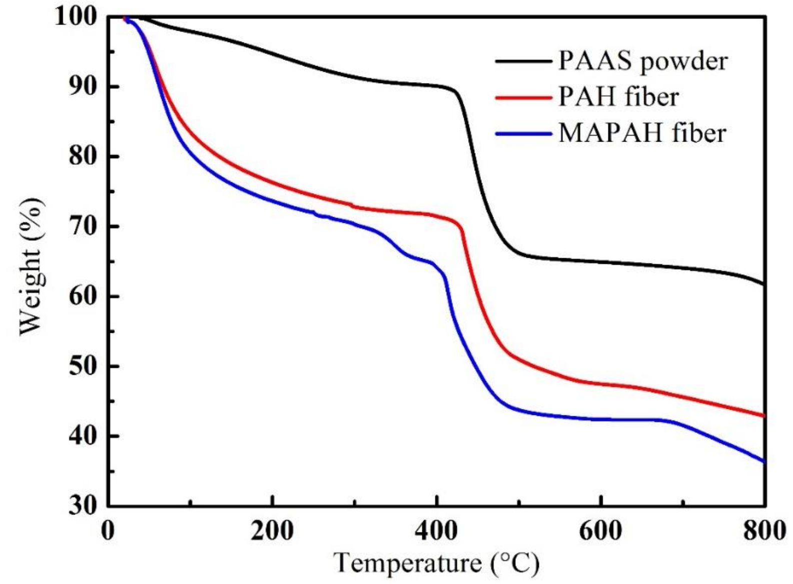

Supplementary Figure 15. TGA curves of the PAAS solid powder, PAH fiber and MAPAH fiber.

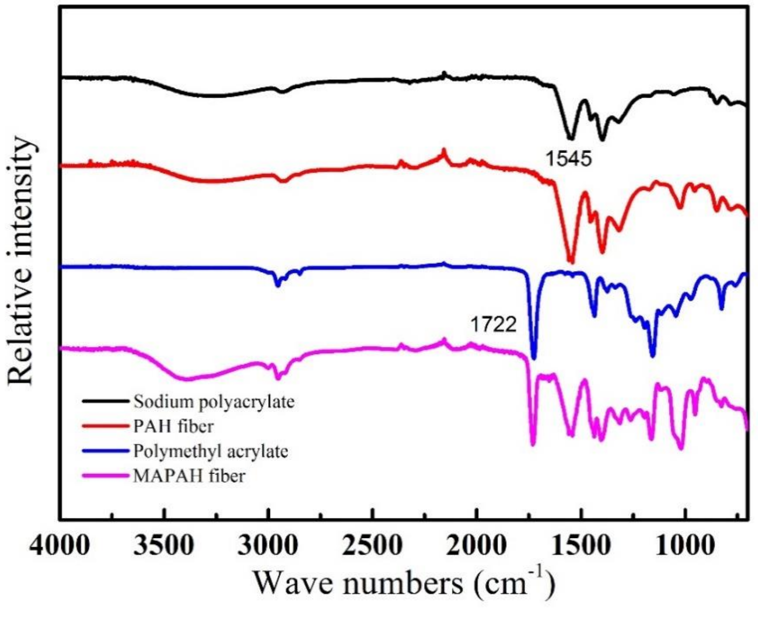

Supplementary Figure 16. Infrared spectra of sodium polyacrylate (PAAS), polymethyl acrylate (PMA) and freeze-dried PAH and MAPAH fibers.

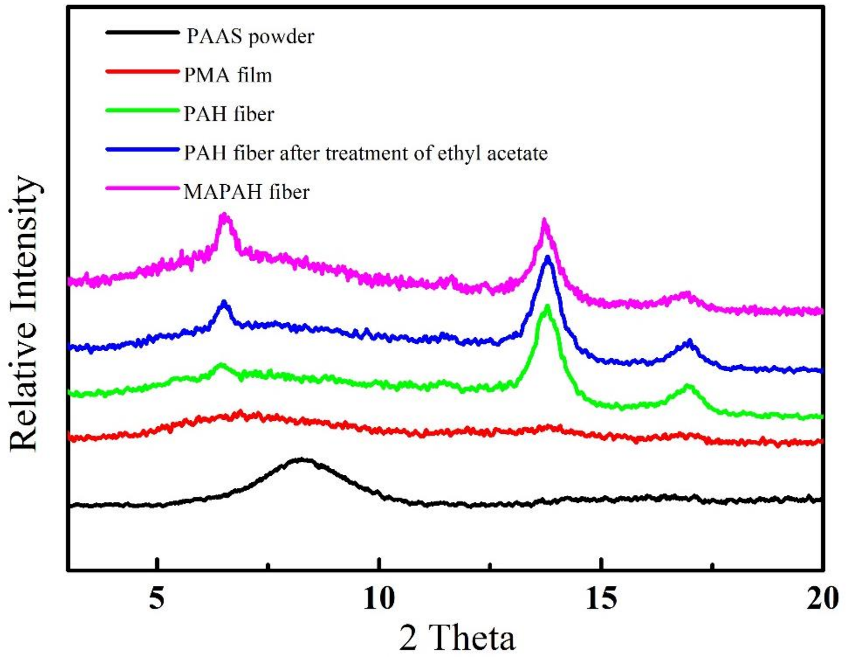

Supplementary Figure 17. Comparison of XRD spectra of different samples.

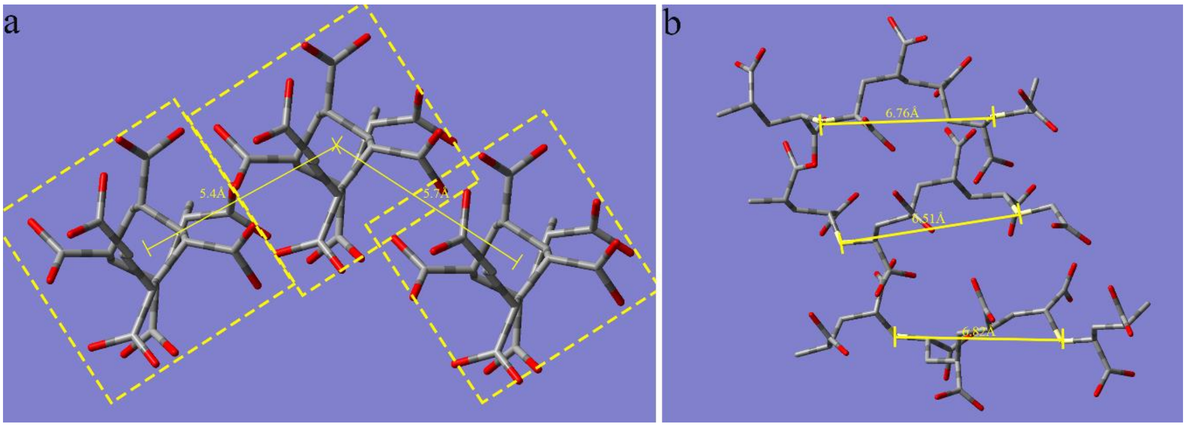

Supplementary Figure 18. Molecular mechanics simulation of the alignment structure of PAAS chains. For clarity, only carbon (grey) and oxygen (red) atoms are shown. The three PAAS chains are roughly parallel to each other. For each PAAS chain, it takes a α-helix-like conformation, which has a pitch around 6.5 Å. The average distance between the center of these α-helix is around 5.4 Å.

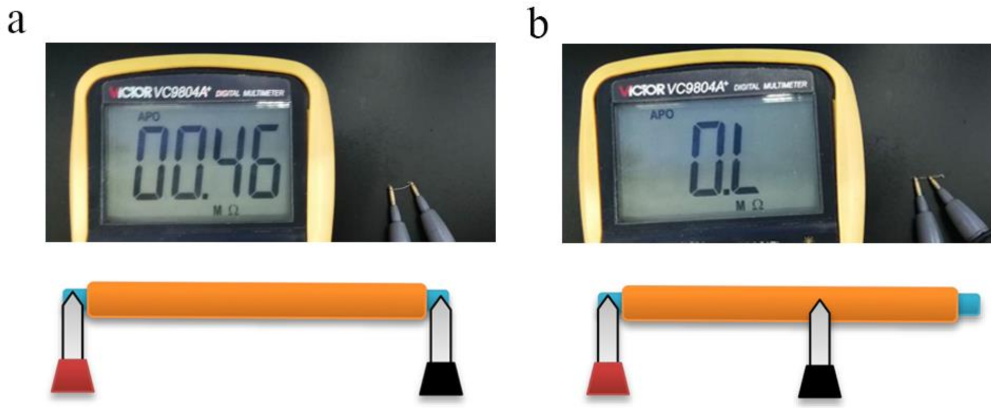

Supplementary Figure 19. Demonstration of the insulation property of the PMA coating layer.

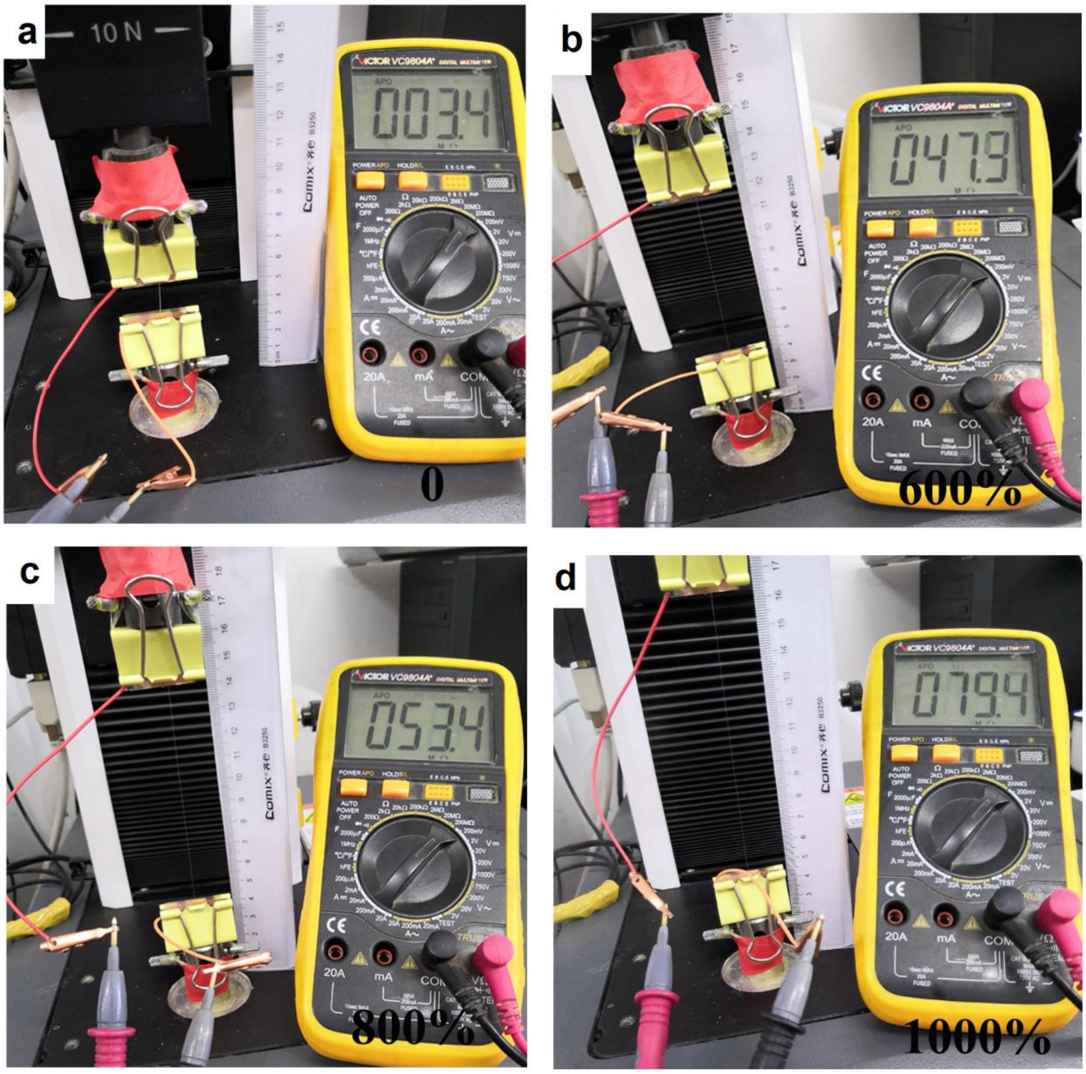

Supplementary Figure 20. Measurement of the electrical resistance of MAPAH fiber at large strains.

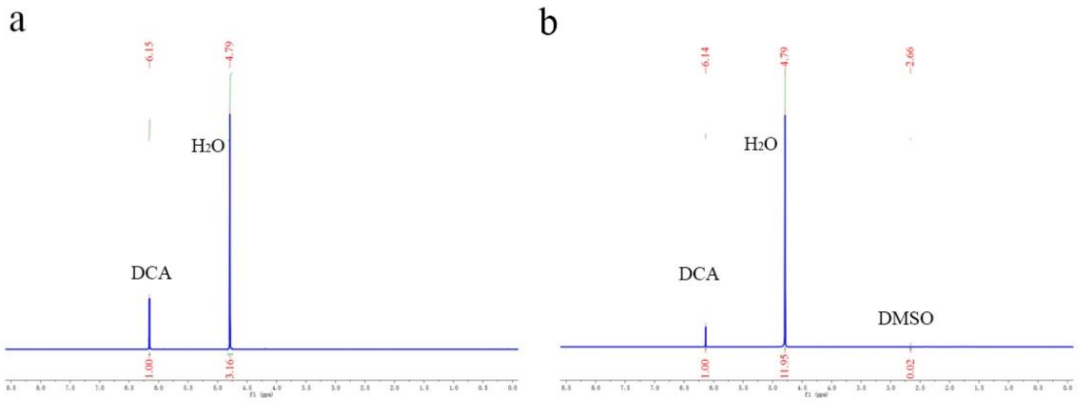

Supplementary Figure 21. ^{1}H NMR of a MAPAH fiber dissolved in D_2O with dichloroacetic acid as internal reference. a) without MAPAH fiber; b) with MAPAH fiber dissolved in D_2O.

Supplementary References

[1] Gaussian 16, Revision B.01, M. J. Frisch, G. W. Trucks, H. B. Schlegel, G. E. Scuseria, M. A. Robb, J. R. Cheeseman, G. Scalmani, V. Barone, G. A. Petersson, H. Nakatsuji, X. Li, M. Caricato, A. V. Marenich, J. Bloino, B. G. Janesko, R. Gomperts, B. Mennucci, H. P. Hratchian, J. V. Ortiz, A. F. Izmaylov, J. L. Sonnenberg, D. Williams-Young, F. Ding, F. Lipparini, F. Egidi, J. Goings, B. Peng, A. Petrone, T. Henderson, D. Ranasinghe, V. G. Zakrzewski, J. Gao, N. Rega, G. Zheng, W. Liang, M. Hada, M. Ehara, K. Toyota, R. Fukuda, J. Hasegawa, M. Ishida, T. Nakajima, Y. Honda, O. Kitao, H. Nakai, T. Vreven, K. Throssell, J. A. Montgomery, Jr., J. E. Peralta, F. Ogliaro, M. J. Bearpark, J. J. Heyd, E. N. Brothers, K. N. Kudin, V. N. Staroverov, T. A. Keith, R. Kobayashi, J. Normand, K. Raghavachari, A. P. Rendell, J. C. Burant, S. S. Iyengar, J. Tomasi, M. Cossi, J. M. Millam, M. Klene, C. Adamo, R. Cammi, J. W. Ochterski, R. L. Martin, K. Morokuma, O. Farkas, J. B. Foresman, and D. J. Fox, Gaussian, Inc., Wallingford CT, **2016**.

[2] (a) A. K. Rappe, K. S. Colwell, C. J. Casewit. *J. Inorg. Chem.* **1993**, *32*, 3438-3450. (b) A. K. Rappe, C. J. Casewit, K. S. Colwell, W. A. Goddard, W. M. Skiff. *J. Am. Chem. Soc.* **1992**, *114*, 10024-10035. (c) A. K. Rappe, C. J. Casewit, K. S. Colwell. *J. Am. Chem. Soc.* **1992**, *114*, 10035-10046. (d) A. K. Rappe, C.J. Casewit, K. S. Colwell. *J. Am. Chem. Soc.* **1992**, *114*, 10046-10053.

[3] J. L. Pascual-Ahuir, E. Silla, and I. Tuñón. *J. Comp. Chem.* **1994**, *15*, 1, 127-1138.
