# Supplementary material for: Bioinspired ultra-stretchable and anti-freezing conductive hydrogel fibers with ordered and reversible polymer chain alignment
Source: Nat Commun. 2018 Sep 4;9:3579. doi: 10.1038/s41467-018-05904-z (PMC6123392; doi:10.1038/s41467-018-05904-z)
Supplement: Supplementary file 2 — Description of Additional Supplementary Files [file 41467_2018_5904_MOESM2_ESM.pdf]

Description of Additional Supplementary Files

File Name: Supplementary Movie 1
Description: The preparation of a PAH fiber from the optimal PAAS solution.

File Name: Supplementary Movie 2
Description: The water sensitivity of a PAH fiber and the water resistance of a MAPAH fiber at stretched status (~ 200% elongation).

File Name: Supplementary Movie 3
Description: The hysteresis effect during a MAPAH fiber's recovery after a large elongation.

File Name: Supplementary Movie 4
Description: A MAPAH fiber serves as a highly stretchable wire in an electric circuit.
